# New *SNCA* mutation and structures of α-synuclein filaments from juvenile-onset synucleinopathy

**DOI:** 10.1007/s00401-023-02550-8

**Published:** 2023-02-27

**Authors:** Yang Yang, Holly J. Garringer, Yang Shi, Sofia Lövestam, Sew Peak-Chew, Xianjun Zhang, Abhay Kotecha, Mehtap Bacioglu, Atsuo Koto, Masaki Takao, Maria Grazia Spillantini, Bernardino Ghetti, Ruben Vidal, Alexey G. Murzin, Sjors H. W. Scheres, Michel Goedert

**Affiliations:** 1 Medical Research Council Laboratory of Molecular Biology, Cambridge, UK; 2 Department of Pathology and Laboratory Medicine, Indiana University School of Medicine, Indianapolis, IN USA; 3 Thermo Fisher Scientific, Eindhoven, The Netherlands; 4 Department of Clinical Neurosciences, University of Cambridge, Cambridge, UK; 5 Yomiuri-Land Keiyu Hospital, Tokyo, Japan; 6 Department of Clinical Laboratory and Internal Medicine, National Center of Neurology and Psychiatry, Tokyo, Japan; 7 Department of Neurology and Brain Bank, Mihara Memorial Hospital, Isesaki, Japan; 8 Present Address: MOE Frontier Science Center for Brain Science and Brain-Machine Integration, Zhejiang University, Hangzhou, China

**Keywords:** α-Synuclein, Duplication mutation in *SNCA*, Juvenile-onset synucleinopathy, Parkinson’s disease, Multiple system atrophy, Cryo-electron microscopy

## Abstract

A 21-nucleotide duplication in one allele of *SNCA* was identified in a previously described disease with abundant α-synuclein inclusions that we now call juvenile-onset synucleinopathy (JOS). This mutation translates into the insertion of MAAAEKT after residue 22 of α-synuclein, resulting in a protein of 147 amino acids. Both wild-type and mutant proteins were present in sarkosyl-insoluble material that was extracted from frontal cortex of the individual with JOS and examined by electron cryo-microscopy. The structures of JOS filaments, comprising either a single protofilament, or a pair of protofilaments, revealed a new α-synuclein fold that differs from the folds of Lewy body diseases and multiple system atrophy (MSA). The JOS fold consists of a compact core, the sequence of which (residues 36–100 of wild-type α-synuclein) is unaffected by the mutation, and two disconnected density islands (A and B) of mixed sequences. There is a non-proteinaceous cofactor bound between the core and island A. The JOS fold resembles the common substructure of MSA Type I and Type II dimeric filaments, with its core segment approximating the C-terminal body of MSA protofilaments B and its islands mimicking the N-terminal arm of MSA protofilaments A. The partial similarity of JOS and MSA folds extends to the locations of their cofactor-binding sites. In vitro assembly of recombinant wild-type α-synuclein, its insertion mutant and their mixture yielded structures that were distinct from those of JOS filaments. Our findings provide insight into a possible mechanism of JOS fibrillation in which mutant α-synuclein of 147 amino acids forms a nucleus with the JOS fold, around which wild-type and mutant proteins assemble during elongation.

**Supplementary Information:**

The online version contains supplementary material available at 10.1007/s00401-023-02550-8.

## Introduction

α-Synuclein is the major component of the filamentous inclusions found in several neurodegenerative diseases, including Parkinson’s disease (PD), dementia with Lewy bodies (DLB) and multiple system atrophy (MSA) [10]. We previously showed that assembled α-synuclein adopts distinct molecular conformers in the MSA and Lewy folds [30, 42]. Most cases of PD and DLB are sporadic but some are inherited in a dominant manner. Gene dosage and missense mutations have been described, with mean ages of onset of disease in the fourth and fifth decades [5, 13, 26, 32, 43].

Some of us previously described a case of synucleinopathy with an age of onset of disease of 13 years and death 2 years later that we named early-onset DLB [37]. The proband developed rapidly progressing Parkinsonism and cognitive impairment. Abundant α-synuclein-positive pathology was present throughout the cerebral cortex and several subcortical nuclei. Severe neuronal loss and gliosis in cerebral cortex and substantia nigra were present alongside vacuolar changes in the upper layers of the neocortex. α-Synuclein pathology exceeded that of typical DLB.

We now report the cryo-EM structures of α-synuclein filaments from the frontal cortex of this case. They are different from the Lewy fold observed in PD and DLB, and share only a partial similarity with the structures of MSA filaments [30]. Since distinct α-synuclein folds characterise different diseases [30, 42], this unique case appears to represent a different disease, which we now name juvenile-onset synucleinopathy (JOS).

We also report the likely cause of JOS: a previously overlooked 21-nucleotide duplication in the second exon of one allele of *SNCA*. This mutation, translating into insertion of MAAAEKT after residue 22 of α-synuclein, probably occurred de novo. Both wild-type and mutant proteins are present in the JOS filaments. Unravelling their structures allowed us to propose a possible mechanism of fibrillation, in which the filaments are nucleated by the longer mutant α-synuclein and elongated through incorporation of both wild-type and mutant proteins. Testing this hypothesis through in vitro assembly of recombinant wild-type α-synuclein, the insertion mutant and their mixture was not possible, because the resulting filaments had different structures from those of JOS filaments.

## Materials and methods

### Clinical history and neuropathology

The case with JOS has been reported [37]. A 13-year-old individual developed rapidly progressive Parkinsonism, cognitive impairment, dysarthria and myoclonus, and died two years later. The number of α-synuclein deposits exceeded that of typical DLB.

### Sequencing of SNCA coding exons

Genomic DNA was extracted from frontal cortex. Coding exons of *SNCA* and flanking intronic sequences were amplified by polymerase chain reaction, screened by agarose gel electrophoresis and sequenced using the dideoxy method. Amplified exon 2 DNA from the JOS case was subcloned into pCR2.1 TA (Invitrogen). Recombinant plasmids were isolated from 48 clones and DNA from 5 of these clones was sequenced in both directions, as described [40].

### Extraction of α-synuclein filaments

Sarkosyl-insoluble material was extracted from frontal cortex of the individual with JOS, as described [38]. In brief, frozen tissues were thawed and homogenised in 20 vol (v/w) extraction buffer consisting of 10 mM Tris–HCl, pH 7.5, 0.8 M NaCl, 10% sucrose and 1 mM EGTA. Homogenates were brought to 2% sarkosyl and incubated for 30 min at 37 °C. Following a 10 min centrifugation at 10,000 g, the supernatants were spun at 100,000 g for 20 min. Pellets were resuspended in 500 µl/g extraction buffer and centrifuged at 3,000 g for 5 min. Supernatants were diluted three-fold in 50 mM Tris–HCl, pH 7.5, containing 0.15 M NaCl, 10% sucrose and 0.2% sarkosyl, and spun at 166,000 g for 30 min. Sarkosyl-insoluble pellets were resuspended in 100 µl/g of 20 mM Tris–HCl, pH 7.4.

### Immunolabelling, histology and silver staining

Immunogold negative-stain EM and immunoblotting were carried out as described [9]. Filaments were extracted from the frontal cortex of the individual with JOS. PER4, a rabbit polyclonal serum that was raised against a peptide corresponding to residues 116–131 of human α-synuclein [33], was used at 1:50. Images were acquired at 11,000 × with a Gatan Orius SC200B CCD detector on a Tecnai G2 Spirit at 120 kV. For immunoblotting, samples were resolved on 4–12% Bis–Tris gels (NuPage) and primary antibodies were diluted in PBS plus 0.1% Tween 20 and 5% non-fat dry milk. Before blocking, membranes were fixed with 1% paraformaldehyde for 30 min. Primary antibodies were: Syn303 [a mouse monoclonal antibody that recognises residues 1–5 of human α-synuclein [8]] (BioLegend) at 1:4000, Syn1 [a mouse monoclonal antibody that recognises residues 91–99 of human α-synuclein [25]] (BD Biosciences) at 1:4000 and PER4 at 1:4000. Histology and immunohistochemistry were carried out as described [31, 35]. Following deparaffinisation, the sections (8 µm) underwent heat-induced antigen retrieval in 10 mM citrate buffer, pH 6.0. The primary anti-α-synuclein antibodies were: Syn1 (1:250), 4B12 [a mouse monoclonal antibody that recognises residues 103–108 of human α-synuclein [14]] (BioLegend) at 1:250, MJFR14 [a rabbit monoclonal antibody that has been reported to recognise assembled human α-synuclein [17]] (Abcam) at 1:1000 and anti-pS129 [a rabbit monoclonal antibody specific for α-synuclein phosphorylated at S129] (Abcam) at 1:800. Anti-Iba1 (Antibodies.com) and anti-GFAP antibodies (Dako), which recognise microglia and astrocytes, respectively, were used at 1:200. For signal detection, we used ImmPress HRP anti-rabbit/mouse IgG and ImmPACT SG kits (Vector Laboratories) or Alexa fluorophore-conjugated secondary antibodies (Invitrogen) at 1:250. Luminescent conjugated oligothiophene pFTAA (pentameric form of formyl thiophene acetic acid) was used at 3 µM, as described [16]. To visualise inclusions, sections were also silver-impregnated according to Gallyas-Braak [3, 7]. Some sections were counterstained with nuclear fast red or haematoxylin.

### Mass spectrometry

Sarkosyl-insoluble pellets were resuspended in 100 µl HFIP [hexafluoroisopropanol]. Following a 3 min sonication at 50% amplitude (QSonica), they were incubated at 37 °C for 2 h and centrifuged at 100,000 g for 15 min, before being dried by vacuum centrifugation. They were resuspended in 4 M urea and 50 mM ammonium bicarbonate (ambic), before being reduced with 5 mM dithiothreitol at 37 °C for 30 min and alkylated in the dark at room temperature for 30 min with 10 mM iodoacetamide. LysC (Promega) was then added to the samples for 2 h at 25 °C. They were diluted to 1.5 M urea with 50 mM ambic and incubated with trypsin (Promega) at 30 °C overnight. Digestion was stopped by the addition of formic acid to 0.5%, followed by centrifugation at 16,000 g for 5 min. The supernatants were desalted using in-house-made C18 stage tips (3 M Empore) packed with Poros oligo R3 (Thermo Scientific) resin. Bound peptides were eluted stepwise with 30, 50 and 80% acetonitrile in 0.5% formic acid and partially dried in a SpeedVac (Savant). The peptide mixtures were analysed by LC–MS/MS using a Q Exactive Plus hybrid quadrupole-Orbitrap mass spectrometer, coupled to an Ultimate 3000 RSLC nano System (Thermo Fisher Scientific). Peptides were trapped by a 100 µm × 2 cm PepMap100 C18 nano trap column (Thermo Fisher Scientific) and separated on a 75 µm × 25 cm, nanoEase M/Z HSS C18 T3 column (Waters) using solvents consisting of buffers A (2% acetonitrile, 0.1% formic acid) and B (80% acetonitrile, 0.1% formic acid) at a flow rate of 300 nl/min. The collected data were processed using MaxQuant with the integrated Andromeda search engine (v.2.1.3.0). MS/MS spectra were searched against the Uniprot *Homo sapiens* refProteome. Carbamidomethylation of cysteines was set as fixed modification, while methionine oxidation and N-terminal protein acetylation were considered to be variable. Enzymatic specificity of trypsin for up to two missed cleavages was allowed. Using the visualisation tap of maxQuant, MS/MS spectra of wild-type and inserted sequences of α-synuclein were selected and manually checked to confirm the insertion site.

### Expression, purification and assembly of recombinant α-synuclein

Wild-type and mutant human α-synucleins were expressed and purified using modifications of a published protocol [20, 23]. Constructs were made via in-fusion PCR cloning using human α-synuclein in pRK172. Expression was carried out in *E. coli* BL21(DE3) cells that were induced with 1 mM IPTG at an OD_600_ of 0.8 for 3–4 h and centrifuged at 2000 g at 4 °C for 20 min. They were resuspended in cold buffer (10 mM Tris–HCl, pH 7.4, 5 mM EDTA, 0.1 mM AEBSF, supplemented with cOmplete EDTA-free protease inhibitor cocktail) and lysed by sonication using a Sonics VCX-750 Vibra Cell Ultrasonic processor, followed by centrifugation at 20,000 g at 4 °C for 45 min. The pellets were discarded and the pH of the supernatants was lowered to 3.5 with HCl, stirred for 30 min at room temperature and centrifuged at 50,000 g at 4 °C for 1 h. The pH was then increased to 7.4 with NaOH and the supernatants loaded onto an ion exchange HiTrap Q HP column and eluted with a 0–1 M NaCl gradient. Fractions containing α-synuclein were precipitated with ammonium sulphate (0.3 g/ml) for 30 min at 4 °C and centrifuged at 20,000 g at 4 °C for 30 min. The pellets were resuspended in PBS and loaded onto a HiLoad 16/60 Superdex (GE Healthcare) column equilibrated in PBS. The purity of α-synuclein was analysed by SDS-PAGE and protein concentrations were determined spectrophotometrically using an extinction coefficient of 5600 M^−1^ cm^−1^. The protein-containing fractions were pooled and concentrated to 3–6 mg/ml. To separate wild-type from mutant α-synuclein, the samples were run on 16% Tricine gels (Invitrogen) and immunoblotted with Syn1. Before assembly, to remove aggregates, wild-type α-synuclein, the insertion mutant and their 1:1 mixture were centrifuged at 2000 g for 10 min at 4 °C and the supernatants used. For assembly, 130 µM recombinant α-synucleins were incubated in microplates (PerkinElmer, Viewplate-384F), with 200 rpm shaking (Fluostar Omega; BMGLabtech) for 1 min every 2 min in 30 µl phosphate-buffered saline (PBS) at 37 °C for 4 days. Prior to further processing, the presence of filaments was verified by negative-stain EM.

### Electron cryo-microscopy

Extracted filaments and assembled fractions were centrifuged at 3000 g for 3 min and applied to glow-discharged holey carbon gold grids (Quantifoil Au R1.2/1.3, 300 mesh), which were glow-discharged with an Edwards (S150B) sputter coater at 30 mA for 30 s. Aliquots of 3 µl were applied to the grids and blotted for approximately 3–5 s with filter paper (Whatman) at 100% humidity and 4 °C, using a Vitrobot Mark IV (Thermo Fisher). Datasets were acquired on Titan Krios G2, G3 and G4 microscopes (Thermo Fisher Scientific) operated at 300 kV. Images of α-synuclein filaments extracted from frontal cortex of the JOS case were obtained using a Falcon-4i detector and a Selectris-X energy filter (Thermo Fisher Scientific) with a slit width of 10 eV to remove inelastically scattered electrons. Images of α-synuclein filaments assembled from recombinant proteins were obtained using a Falcon-4 detector without energy filter or a Gatan K3 detector in super-resolution counting mode, using a Bioquantum energy filter with a slid width of 20 eV. Images were recorded with a total dose of 40 electrons per Å^2^.

### Helical reconstruction

Movie frames were gain-corrected, aligned, dose-weighted and summed into a single micrograph using RELION’s own motion correction program [44]. Contrast transfer function (CTF) parameters were estimated using CTFFIND-4.1 [27]. Subsequent image-processing steps were performed using helical reconstruction methods in RELION [12]. Filaments were picked manually for the JOS dataset; auto-picking was used for the in vitro assembly datasets. Reference-free 2D classification was performed to select suitable segments for generating initial models and for further processing. Initial models were generated de novo from 2D class averages using *relion helix inimodel2d* [29]. Helical twist and rise were optimised during 3D autorefinement. Bayesian polishing and CTF refinement were used to further improve the resolution of reconstructions [45]. Final maps were sharpened using standard post-processing procedures in RELION and their resolutions calculated based on the Fourier shell correlation (FSC) between two independently refined half-maps at 0.143 [28]. Helical symmetry was imposed on the post-processed maps using the *relion helix toolbox* program.

### Model building

Atomic models were built in Coot [4] using shared substructures of MSA Type I and Type II filaments (PDB:6XYO; PDB:6XYQ) as templates. Coordinate refinements were performed using *Servalcat* [41]. Final models were obtained using refinement of the asymmetric unit against the unsharpened half-maps with helical symmetry in *Servalcat*. Model statistics are given in Supplementary Tables 1 and 2.

## Results

### Genetic analysis

**Fig. 1:**
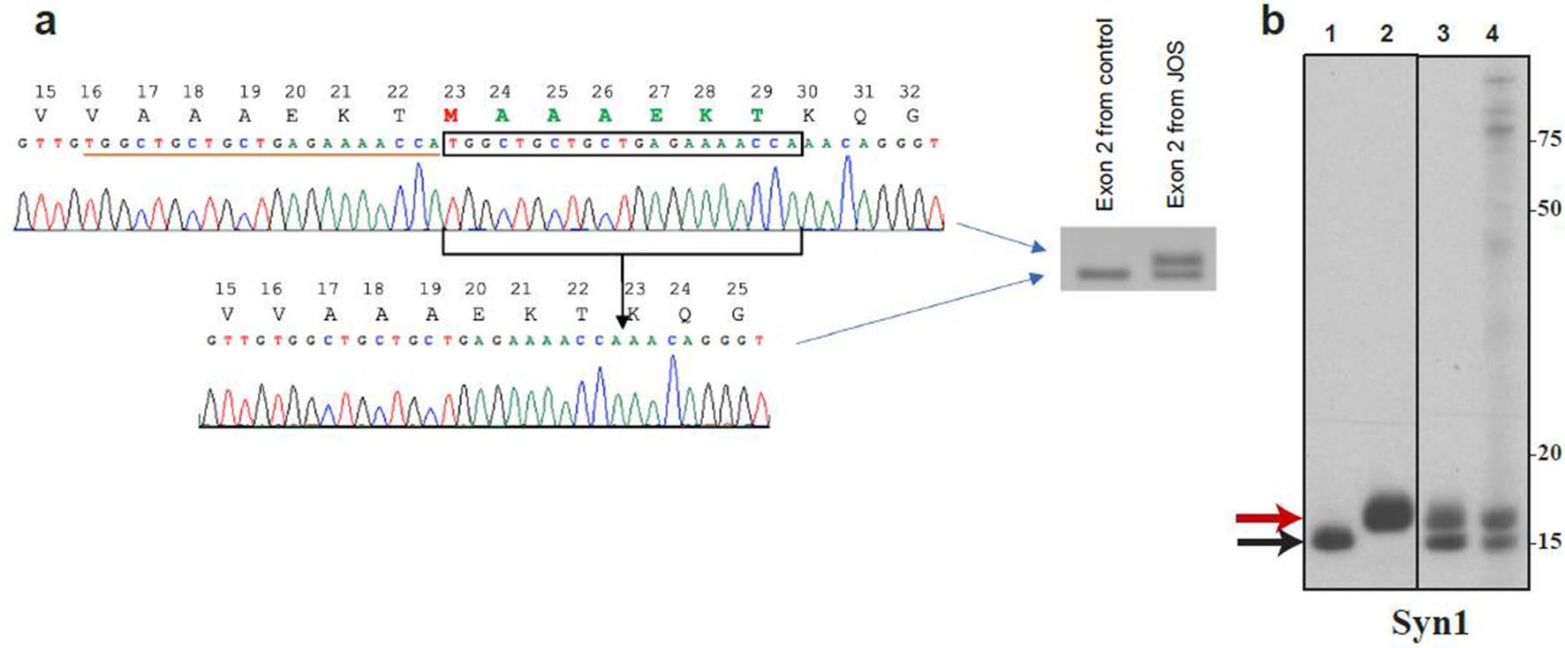
Seven amino acid insertion mutation in JOS α-synuclein. **a** PCR amplification of *SNCA* exon 2 DNA from JOS gave the wild-type band of 377 nucleotides and a band of 398 nucleotides. Sequencing of the latter showed the presence of a 21 bp duplication, resulting in an in-frame insertion of seven amino acids (MAAAEKT) in the predicted protein. **b** Immunoblotting of recombinant wild-type and mutant α-synuclein, and of JOS sarkosyl-insoluble material using Syn1. Lanes: 1, recombinant wild-type α-synuclein; 2, recombinant mutant α-synuclein; 3, mixture of recombinant wild-type and mutant α-synuclein; 4, JOS sarkosyl-insoluble material

PCR amplification of control exon 2 DNA of *SNCA* produces a fragment of 377 nucleotides. Gel electrophoresis of amplified exon 2 DNA from the JOS patient revealed the presence of two fragments, the wild-type band and an additional band of 398 nucleotides (Fig. 1a). DNA sequencing showed a 21-nucleotide duplication (TGGCTGCTGCTGAGAAAACCA) in one allele of *SNCA* (c.47_67dup). In α-synuclein, this translates into the insertion of 7 residues (MAAAEKT) after residue 22, resulting in a protein of 147 amino acids. The PCR products of exon 2 amplification from JOS were subcloned into pCR2.12 and DNA sequencing confirmed the presence of both wild-type and mutant alleles. The mutation probably occurred de novo, since the proband’s parents did not exhibit symptoms of Parkinsonism or cognitive impairment.

### Immunoblotting and mass spectrometry of sarkosyl-insoluble material

Recombinant wild-type (140 amino acids) and mutant (147 amino acids) α-synucleins ran as discrete bands of approximately 15 and 16 kDa on 16% Tricine gels. When mixed together, they gave two separate bands on immunoblots with Syn1. Immunoblots of sarkosyl-insoluble material from the frontal cortex of the JOS individual gave two separate bands of similar intensities that aligned with the bands of recombinant wild-type and mutant α-synucleins (Fig. 1b). Mass spectrometry of the same material confirmed the presence of wild-type and mutant α-synucleins (Figure S1).

### Neuropathology

In cerebral cortex and several subcortical nuclei of JOS, we previously reported the presence of abundant intracytoplasmic α-synuclein inclusions in nerve cell bodies and processes [37]. These findings were confirmed by immunostaining of frontal cortex with anti-α-synuclein antibodies Syn1, 4B12, MJFR14 and pS129 (Figure S2a). α-Synuclein inclusions were also present in a few cells with a smaller diameter, consistent with their presence in some glial cells. The α-synuclein inclusions of JOS were labelled by luminescent conjugated oligothiophene pFTAA (Figure S2b) and were weakly Gallyas-Braak silver-positive (Figure S2c). Triple-labelling immunofluorescence using anti α-synuclein antibody Syn1, as well as anti-Iba1 and anti-GFAP antibodies, showed prominent gliosis in areas with α-synuclein inclusions (Figure S2d). By immunoelectron microscopy of sarkosyl-insoluble material, abundant α-synuclein filaments with a diameter of 10 nm were present (Figure S3a). By immunoblotting with antibodies Syn303, Syn1 and PER4 (Figure S3b), high-molecular weight material, as well as full-length α-synuclein were the predominant species. Truncated α-synuclein was less abundant. None of the antibodies distinguished between wild-type and mutant α-synucleins.

### Structures of α-synuclein filaments from JOS

**Fig. 2:**
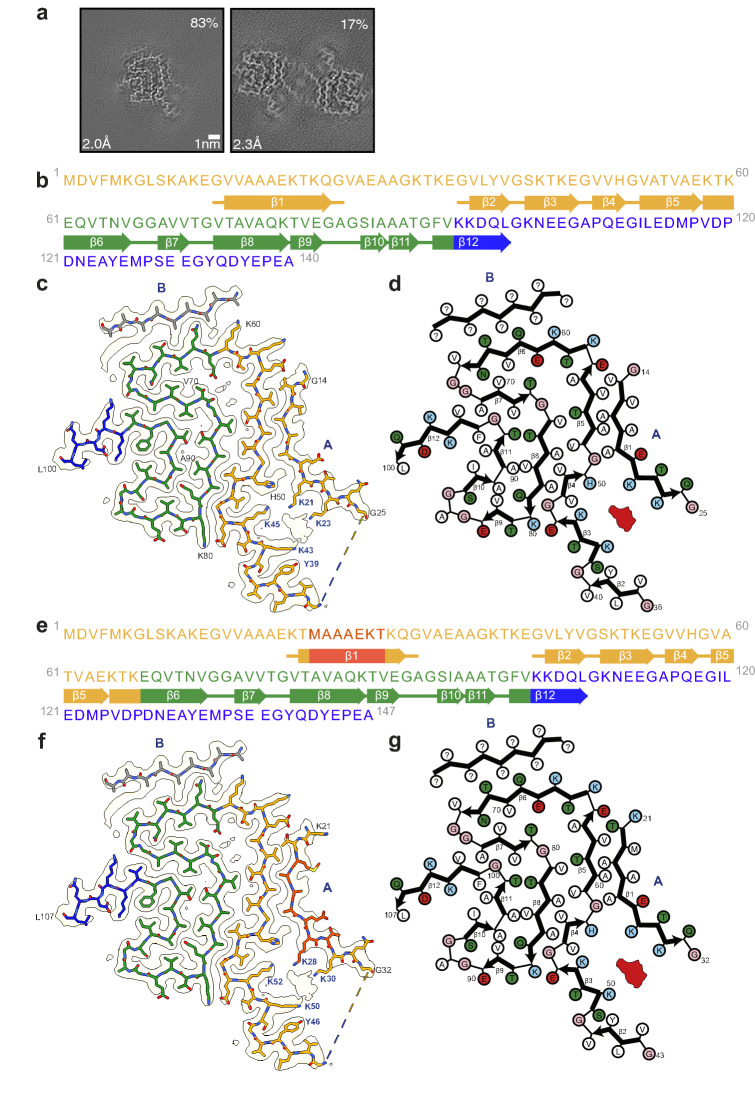
Cryo-EM structures of JOS singlet α-synuclein filaments. **a** Cross-sections through the cryo-EM reconstructions, perpendicular to the helical axis and with a projected thickness of approximately one rung, are shown for α-synuclein singlet and doublet filaments from JOS. Scale bar, 1 nm. **b**, **e** Amino acid sequences of wild-type (**b**) and mutant (**e**) α-synucleins. The N-terminal regions (1–60 or 1–67) are shown in yellow, the non-Abeta component regions (residues 61–95 or 68–102) in green and the C-terminal regions (residues 96–140 or 103–147) in blue. The insert (MAAAEKT) is indicated in red. Thick connecting lines with arrowheads indicate β-strands. **c**, **f** Unsharpened cryo-EM density map and atomic model of the JOS fold with wild-type (**c**) or mutant (**f**) α-synuclein. Island B is indicated in grey. Putative cofactor densities are shown in red. **d**, **g** Schematics of JOS fold with wild-type (**d**) or mutant (**g**) α-synuclein. Negatively charged residues are in red, positively charged residues in blue, polar residues in green, non-polar residues in white, sulfur-containing residues in yellow and glycines in pink. Thick connecting lines with arrowheads indicate β-strands. Unknown residues are indicated by question marks. Putative cofactor densities are shown in red

**Fig. 3:**
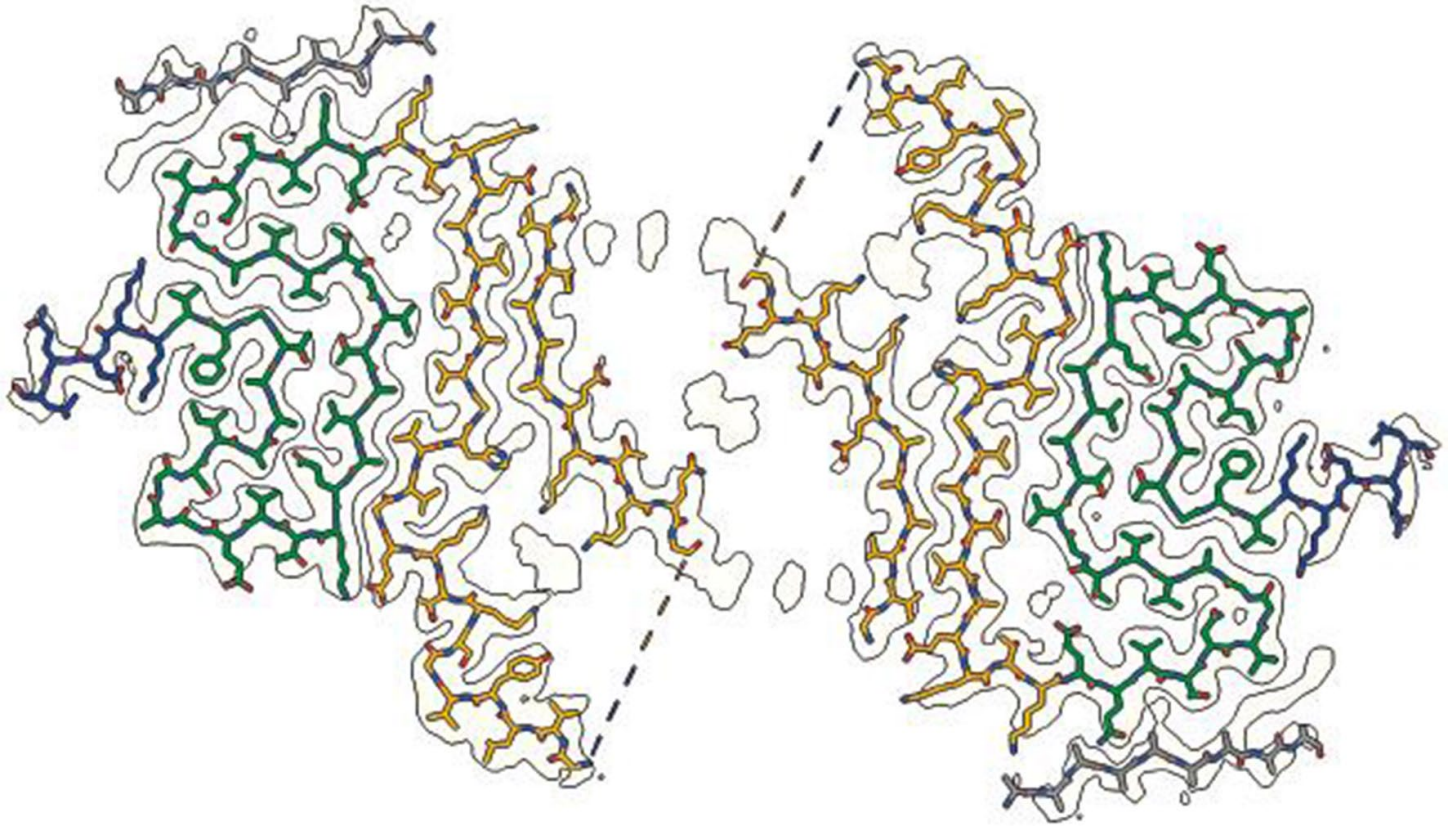
Cryo-EM structures of JOS doublet α-synuclein filaments. Cryo-EM density map and atomic model of doublet filaments coloured as in Fig. 2

The vast majority of α-synuclein filaments from the frontal cortex of the individual with JOS (83%) comprise a single protofilament. The structure of these filaments was determined at 2.0 Å resolution and revealed a new fold that we call the JOS fold. In the structure of the remaining α-synuclein filaments (17%), determined at 2.3 Å resolution, there are two identical protofilaments with the JOS fold that pack against each other with C2 symmetry (Figs. 2, 3). Both singlet and doublet filaments have a left-handed helical twist, as established from the densities for backbone oxygen atoms in the cryo-EM maps (Figure S4).

The JOS fold consists of a compact core, spanning residues 36–100 (in the numbering of wild-type α-synuclein), and two disconnected density islands (A and B). There is also a large non-proteinaceous density between the core and island A that may correspond to a bound cofactor and is similar in size and environment to the unidentified densities of Lewy and MSA folds [30, 42]. An additional density, next to the side chain of H50, may represent a post-translational modification. Other densities probably correspond to solvent molecules.

The core sequence is unaffected by the mutation and has virtually the same conformation for residues K43-Q99 as the corresponding segment of MSA protofilament IIB2 (PDB:6XYQ) [30]. The conformation of this segment, which is stabilised in part by a characteristic salt bridge between residues E46 and K80, is also similar to the conformations of wild-type α-synuclein filaments formed following seeded aggregation using MSA brain seeds (PDB:7NCK) [20] and filaments assembled in vitro from H50Q α-synuclein (PDB:6PEO) [1] or A53T α-synuclein (PDB:6LRQ) [36], with minor differences in the inversion of side chain orientations of residues K58 and T59 (Figure S5a).

In the core, most side chain densities are fully resolved in the 2.0 Å resolution map of JOS singlet filaments. In the islands, however, the side chain densities are not well resolved, precluding unambiguous sequence assignment. Since their main chain densities are fully resolved, we attribute the lack of distinct side chain densities to the islands consisting of a mixture of different sequences. These sequences probably come mainly from the amino- and/or carboxy-terminal regions of the same α-synuclein chain, but the incorporation of exogenous peptide fragments cannot be excluded.

In the structure of island A, there are at least three clues that point to which sequences may be incorporated. Island A makes a curved β-strand, the amino-terminal half of which is tightly packed against residues A53 and V55 of the core, whereas its carboxy-terminal half bends away opposite residue H50 and faces the non-proteinaceous density, separating it from residues K43 and K45. Its interface with the core resembles part of the protofilament interface of MSA filaments between the N-terminal arm of protofilaments A and the C-terminal body of protofilaments B. The MSA protofilament interface harbours a non-proteinaceous density that is surrounded by four lysine residues in approximately the same location, suggesting that the residues in island A that face a similar density in the JOS fold are also lysines. A residue with a small side chain, which is packed between A53 and V55, is likely alanine. Side chain densities for Cβ atoms suggest that island A comprises ten consecutive non-glycine residues. Only two α-synuclein sequences satisfy all three clues: _14_GVVAAAEKTKQG_25_ in the 140-residue wild-type protein (Fig. 2c–d) and _21_KTMAAAEKTKQG_32_ in the 147-residue insertion mutant protein (Fig. 2f–g).

There are insufficient clues to suggest which sequences the shorter island B may be made of. However, our assignment of island A with the insertion mutant leaves an N-terminal segment that is long enough to reach and fill up the island B density. A model of the JOS fold made only of mutant protein, in which island B is assigned the sequence _6_KGLSKAKE_13_ and is connected to _21_KTMAAAEKTKQG_32_ of island A, is shown in Figure S6. In this model, residues 13–20, which connect islands B and A, mimic the conformation of the N-terminal arm of MSA protofilaments A. The absence of density for these residues in the cryo-EM map indicates that they are not ordered in the JOS filaments. An alternative explanation for the density of island B is that it can be reached from the last ordered residue of the core (L100) and that it consists of sequences from the C-terminal region of α-synuclein.

The cryo-EM reconstructions of JOS doublet filaments show that island A is essential for dimerisation, with the islands from both protofilaments facing each other across the filament axis. The 2.3 Å resolution map of the doublet filament further supports our sequence assignments for island A. Interestingly, there are no direct contacts between the ordered residues of both protofilaments. Like in dimeric filaments of TMEM106B [31], an additional disconnected density of unknown identity resides on the symmetry axis and mediates filament interactions (Fig. 3). This non-proteinaceous density, which is co-ordinated by neutral polar residues Q24 (Q31 in mutant α-synuclein) from both protofilaments, is probably of a different chemical nature than the large non-proteinaceous densities within each protofilament. The density of island B, which is farthest from the doublet filament axis, is less resolved than in the singlet filament map, but is similar in overall shape.

### Structures of recombinant α-synuclein filaments

**Fig. 4:**
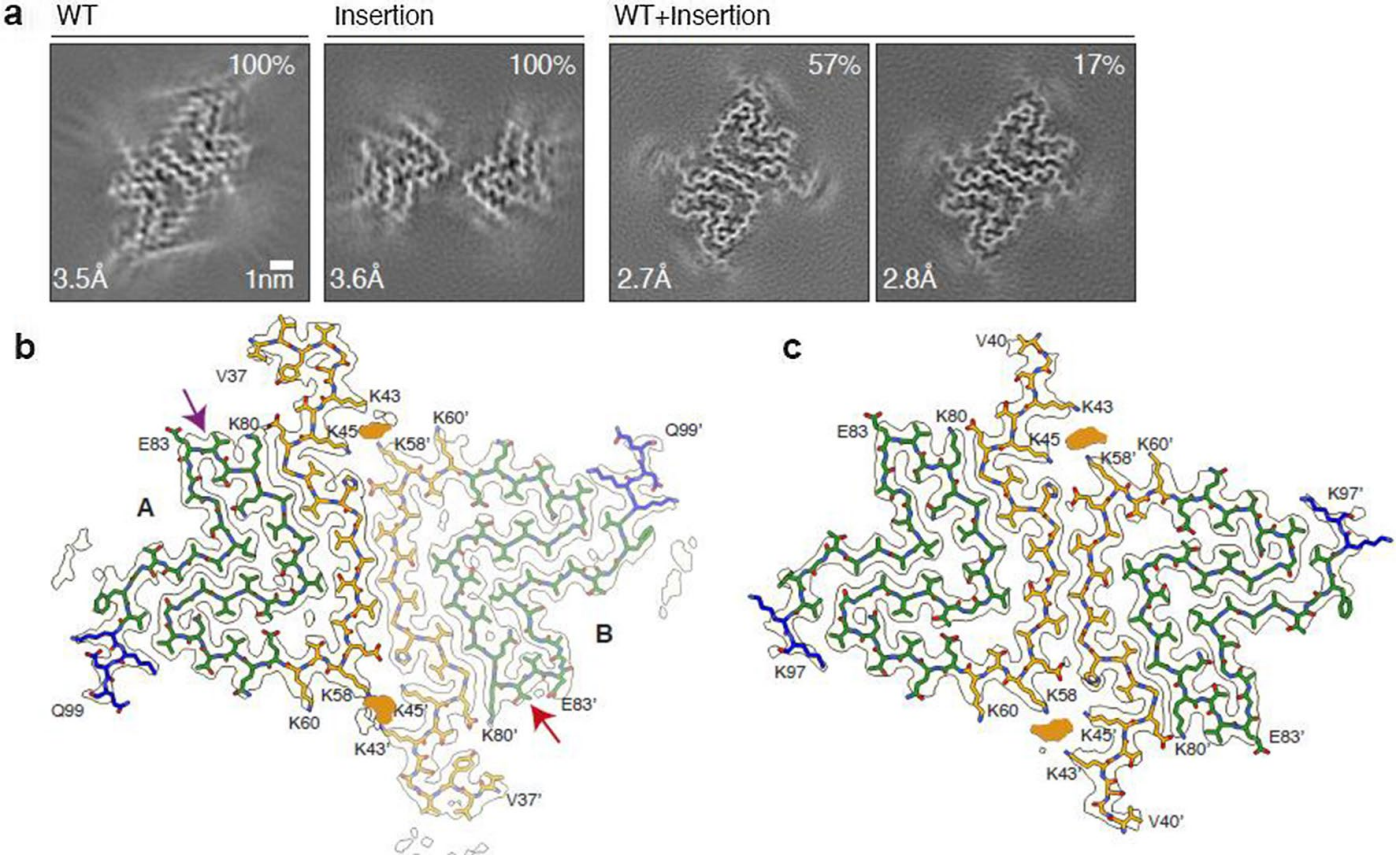
Cryo-EM structures of recombinant α-synuclein filaments. **a** Cross-sections of the cryo-EM reconstructions, perpendicular to the helical axis and with a projected thickness of approximately one rung. From left to right: wild-type α-synuclein; insertion mutant; mixture, showing majority (Type 1) and minority (Type 2) filaments. Scale bar, 1 nm. **b**, **c** Cryo-EM density maps and atomic models of the folds of recombinant protofilaments from 1:1 mixture of wild-type and mutant α-synuclein. The models are coloured as in Fig. 2. Putative phosphate densities are shown in orange. b, The majority of Type 1 filaments comprising two non-identical protofilaments, A (full colours) and B (light colours). The different turns are indicated by purple and red arrows. **c** The minority of Type 2 filaments comprising two identical protofilaments

The in vitro assembly of recombinant human wild-type α-synuclein, the 7-amino acid insertion mutant and a 1:1 mixture of both, yielded filaments that did not reproduce the structures of those from frontal cortex of the JOS case (Fig. 4).

Filaments of wild-type recombinant α-synuclein were of a single type (Fig. 4a). Solved at 3.5 Å resolution, their structures closely resemble known structures of wild-type recombinant α-synuclein filaments (PDB:6H6B; PDB:6CU7) [11, 18] and have minor differences with two others (PDB:6A6B; PDB:6OSJ) [19, 24]. Filaments made of the insertion mutant were also of a single type, but their structure at 3.6 Å resolution was different from that of wild-type α-synuclein filaments (Fig. 4a). This type resembled most closely one of the filaments from in vitro seeded aggregation of wild-type recombinant α-synuclein with seeds of MSA filaments (PDB 7NCG) [20] The same protofilament fold was first observed for filaments assembled from recombinant E46K α-synuclein (PDB:6UFR) [2]. This fold, which comprises residues G36-D98 of wild-type α-synuclein, lacks the N-terminal sequence with the JOS insertion.

Filaments made from a 1:1 mixture of wild-type and mutant human α-synucleins were of at least three Types. We solved the structures of two Types, with the third lacking the twist necessary for helical reconstruction (Fig. 4b, c; Figure S6). The structures were similar in appearance to those of recombinant wild-type α-synuclein and had a left-handed helical twist. The structure of the majority filament (Type 1, 57% of total) was solved to 2.7 Å resolution and revealed two similar, but non-identical, protofilaments labelled A and B. The ordered core of protofilament A spans residues L38-D98, with amino acids L38-G93 adopting almost the same fold as the corresponding segment in MSA protofilament IA (PDB:6XYO) [30] (Figure S5b). The ordered core of protofilament B extends from G36-D98. Both protofilaments differ at the turn K80-A85, with a reversal of inside/outside orientations of the side chains of T81 and V82, and a shift of the segment A85-D98 along the filament axis by one rung. It is not feasible to attribute the distinct protofilament folds to the differences between wild-type and mutant α-synucleins, because the ordered cores are composed of sequences present in both.

We also observed two non-proteinaceous densities in pseudo-symmetrical locations that were surrounded by the side chains of K43 and K45 from one protofilament and K58 and K60 from the other, and vice versa. They probably correspond to phosphate ions from the buffer and are similar in structure to the densities located between the JOS core and island A. JOS segment K43-E83 aligns well with the corresponding segment of protofilament B, resulting in the superposition of both structures, in which residues I88-K97 of JOS structurally align with residues A85-G93 of protofilament B. JOS island A structurally aligns with residues H50-Q62 of protofilament A, with K21 and K23 of the former superimposing on K58 and K60 of the latter (Figure S5c).

The structure of the minority filaments (Type 2, 17% of total) was solved to 2.8 Å resolution. Two identical protofilaments are linked by pseudo-2_1_ symmetry. The ordered protofilament core spans residues G41-V95 and adopts a similar fold to that of protofilament A of Type 1 filament, with minor differences in the relative positions of their amino- and carboxy-terminal parts. Two putative phosphate densities were also present at the inter-protofilament interface (Fig. 4c).

## Discussion

We report a duplication of 21 nucleotides in one allele of *SNCA* in a case of JOS, which inserts the sequence MAAAEKT after residue 22 of α-synuclein. This is the first insertion mutation in *SNCA*. It was not present in the 141,456 individuals of the gnomAD project [15]. The clinical picture was more severe than in DLB and the age of onset (13 years) was atypical. Previously, missense mutations in the coding region of *SNCA* were reported in inherited cases of PD and DLB [26, 43]. Gene dosage mutations (duplications and triplications) of *SNCA* have also been described [5, 13, 32]. Typically, individuals with these mutations have a family history of synucleinopathy. The individual with JOS had no family history, suggesting that the mutation occurred de novo.

Extensive nerve cell loss and abundant filamentous α-synuclein inclusions [37] were confirmed and the latter were weakly Gallyas-Braak silver-positive. α-Synuclein inclusions of MSA are strongly Gallyas-Braak silver-positive, whereas those of PD and DLB are negative [39]. Almost all α-synuclein inclusions were present in what appeared to be neurons in the JOS brain. Luminescent conjugated oligothiophene pFTAA labelled α-synuclein inclusions from the frontal cortex of the JOS individual, similar to what has been described in PD and MSA [16]. As reported previously [37], by negative stain immuno-EM, these filaments had the same staining characteristics as those of PD, DLB and MSA [6, 33, 34]. However, by cryo-EM, a different fold was present.

Immunoblotting and mass spectrometry of sarkosyl-insoluble material showed that both wild-type and mutant α-synucleins make up the JOS filaments. It is conceivable that mutant α-synuclein forms a nucleus, by which wild-type and mutant proteins assemble during elongation, giving rise to mixed filaments. This is reminiscent of mutation R406W in tau, where almost equal amounts of wild-type and mutant tau were present in the sarkosyl-insoluble fractions [22].

We also determined the cryo-EM structures of filaments made from in vitro assembly of recombinant wild-type α-synuclein, the insertion mutant and a 1:1 mixture of both. However, the structures of these filaments were different from those of JOS; additional in vitro studies could therefore not provide meaningful information about mechanisms underlying the formation of JOS filaments in human brain. It is possible that unknown factors, such as post-translational modifications of α-synuclein and/or non-proteinaceous cofactors, are required for formation of the JOS, Lewy and MSA folds. Similar to what has been done for tau [21], it will be important to develop conditions that permit the assembly of recombinant α-synuclein into filaments with identical structures to those from human brains.

The structures of filaments assembled from recombinant wild-type α-synuclein or its insertion mutant were indistinguishable from some of the previously determined structures of recombinant α-synuclein filaments, where two identical protofilaments pack symmetrically [11, 18, 20]. In contrast, assembling wild-type and mutant proteins together, resulted in a majority of filaments with two distinct protofilaments that are packed asymmetrically. As the ordered cores of the protofilaments do not contain the additional 7 residues of the mutant, it is not clear how the 1:1 mixture of wild-type and mutant proteins can lead to asymmetry between protofilaments.

**Fig. 5:**
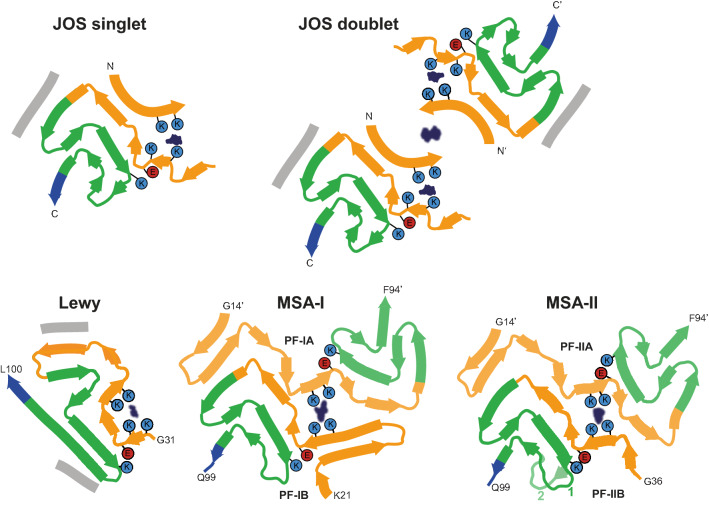
JOS, Lewy and MSA folds of α-synuclein from human brains Schematics of secondary structure elements in JOS, Lewy and MSA folds, depicted as single rungs, and coloured as in Fig. 2. Potential co-factor densities are indicated in dark blue. The positions of their surrounding residues, as well as the supporting salt bridges between E46 and K80 in JOS and MSA folds, and between E35 and K80 in the Lewy fold, are highlighted with coloured circles. Residue numbers with apostrophes indicate those from the other protofilament

The JOS fold is the third known fold of assembled α-synuclein from human brain, the other two being the MSA and Lewy folds (Fig. 5) [30, 42]. The JOS fold differs from the Lewy fold, but it resembles the common substructure of MSA Type I and Type II filaments, with its core segment adopting nearly the same structure as the C-terminal body of MSA protofilaments B and their islands mimicking the N-terminal arms of MSA protofilaments A. This similarity of JOS and MSA folds extends to the locations of their cofactor-binding sites. In dimeric filaments of MSA, K58 of protofilament B makes a salt bridge with E35 of protofilament A. There is a second salt bridge between K60 of protofilament B and E28 of protofilament A. In our model of JOS filaments that are only made of mutant α-synuclein, there are equivalent salt bridges between K65 and E20, and between K67 and E13, which stabilise the interaction between the filament core and the N-terminal region. This suggests that mutant α-synuclein may be prone to form filament nuclei, possibly affected by the presence of a cofactor, that then could grow by incorporation of both wild-type and mutant proteins. Future experiments, possibly including in vitro assembly with recombinant α-synuclein in the presence of relevant cofactors and/or post-translational modifications, will be required for establishing the molecular mechanisms that lead to α-synuclein assembly in JOS.

## Supplementary Information

Below is the link to the electronic supplementary material.

Supplementary file1 (PDF 2044 KB)

## Data Availability

Cryo-EM maps have been deposited in the Electron Microscopy Data Bank (EMDB) with the accession numbers EMD-16188, EMD-16189, EMD-16600, EMD-16603, EMD-16604 and EMD-16608. Corresponding refined atomic models have been deposited in the Protein Data Bank (PDB) under accession numbers 8BQV, 8BQW, 8CE7 and 8CEB. Please address requests for materials to the corresponding authors.
