# Supplementary material for: New SNCA mutation and structures of α-synuclein filaments from juvenile-onset synucleinopathy
Source: Acta Neuropathol. 2023 Feb 27;145(5):561–72. doi: 10.1007/s00401-023-02550-8 (PMC10119069; doi:10.1007/s00401-023-02550-8)
Supplement: Supplementary file 1 — Supplementary file1 (PDF 2044 KB) [file 401_2023_2550_MOESM1_ESM.pdf]

NEW *SNCA* MUTATION AND STRUCTURES OF α-SYNUCLEIN FILAMENTS FROM JUVENILE-ONSET SYNUCLEINOPATHY

Yang Yang[1+], Holly J. Garringer[2+], Yang Shi[1+§], Sofia Lövestam[1], Sew Peak-Chew[1], Xianjun Zhang[3], Abhay Kotecha[3], Mehtap Bacioglu[4], Atsuo Koto[5], Masaki Takao[6,7], Maria Grazia Spillantini[4], Bernardino Ghetti[2], Ruben Vidal[2], Alexey G. Murzin[1*], Sjors H.W. Scheres[1*], Michel Goedert[1*]

1. Medical Research Council Laboratory of Molecular Biology, Cambridge, UK
2. Department of Pathology and Laboratory Medicine, Indiana University School of Medicine, Indianapolis, IN, USA
3. Thermo Fisher Scientific, Eindhoven, The Netherlands
4. Department of Clinical Neurosciences, University of Cambridge, Cambridge, UK
5. Yomiuri-Land Keiyu Hospital, Tokyo, Japan
6. Department of Clinical Laboratory and Internal Medicine, National Center of Neurology and Psychiatry, Tokyo, Japan
7. Department of Neurology and Brain Bank, Mihara Memorial Hospital, Isesaki, Japan

§ Present address: MOE Frontier Science Center for Brain Science and Brain-machine Integration, Zhejiang University, Hangzhou, China

[+]Equal contributions

[*]Corresponding authors scheres@mrc-lmb.cam.ac.uk and mg@mrc-lmb.cam.ac.uk

SUPPLEMENTARY TABLES

Table S1. Cryo-EM data acquisition and structure determination from JOS

	JOS fold (singlet)<br>PDB 8BQV<br>EMD-16188	JOS fold (doublet)<br>PDB 8BQW<br>EMD-16189
**Data acquisition**		
Electron gun	CFEG	CFEG
Detector	Falcon 4i	Falcon 4i
Energy filter slit (eV)	10	10
Magnification	165,000	165,000
Voltage (kV)	300	300
Electron dose (e–/Å^2)	40	40
Defocus range (µm)	0.6 to 1.4	0.6 to 1.4
Pixel size (Å)	0.727	0.727
**Map refinement**		
Symmetry imposed	C1	C2
Initial particle images (no.)	354269	354269
Final particle images (no.)	214769	45038
Map resolution (Å)	2	2.3
FSC threshold	0.143	0.143
Helical twist (°)	-1.33	-1.11
Helical rise (Å)	4.77	4.71
**Model Refinement**		
Model resolution (Å)	2	2.3
FSC threshold	0.5	0.5
Map sharpening *B* factor (Å^2)	-33	-43
Model composition		
Non-hydrogen atoms	1719	3477
Protein residues	252	516
Ligands	0	0
B factors (Å^2)		
Protein	35.5	43.9
R.m.s. deviations		
Bond lengths (Å)	0.0025	0.0068
Bond angles (°)	0.64	1.27
Validation		
MolProbity score	1.82	1.64
Clashscore	7.20	3.39
Poor rotamers (%)	0	0
Ramachandran plot		
Favored (%)	93.59	91.25
Allowed (%)	6.41	8.75
Disallowed (%)	0	0

Table S2. Cryo-EM data acquisition and structure determination of recombinant α-synuclein

	Recombinant wt and mutant α-synuclein (Type 1) PDB 8CE7 EMD-16600	Recombinant wt and mutant α-synuclein (Type 2) PDB 8CEB EMD-16603	Recombinant wt α-synuclein EMD-16608	Recombinant mutant α-synuclein EMD-16604
**Data acquisition**				
Electron gun	CFEG	CFEG	XFEG	XFEG
Detector	Falcon 4	Falcon 4	K3	K3
Energy filter slit (eV)	-	-	20	20
Magnification	96,000	96,000	105,000	105,000
Voltage (kV)	300	300	300	300
Electron dose (e–/Å^2)	40	40	40	40
Defocus range (μm)	-1.0 ~ -2.5	-1.0 ~ -2.5	-1.0 ~ -2.4	-1.0 ~ -2.6
Pixel size (Å)	0.824	0.824	0.73	0.73
**Map refinement**				
Symmetry imposed	C1	C1	C1	C1
Initial particle images (no.)	419,444	419,444	667,633	717,415
Final particle images (no.)	178,778	53,703	249,929	157,337
Map resolution (Å)	2.7	2.8	3.5	3.6
FSC threshold	0.143	0.143	0.143	0.143
Helical twist (°)	-1.58	-179.56	-0.94	-0.93
Helical rise (Å)	4.76	2.38	4.82	4.79
**Model Refinement**				
Model resolution (Å)	2.7	2.8		
FSC threshold	0.5	0.5		
Map sharpening *B* factor (Å^2)	-67	-55		
Model composition				
Non-hydrogen atoms	3504	3152		
Protein residues	504	464		
Ligands	0	0		
B factors (Å^2)				
Protein	60	59		
R.m.s. deviations				
Bond lengths (Å)	0.0064	0.0075		
Bond angles (°)	1.28	1.54		
Validation				
MolProbity score	2.47	2.66		
Clashscore	8.78	7.85		
Poor rotamers (%)	0	0		
Ramachandran plot				
Favored (%)	87.70	86.61		
Allowed (%)	12.30	13.39		
Disallowed (%)	0	0		

SUPPLEMENTARY FIGURES

a

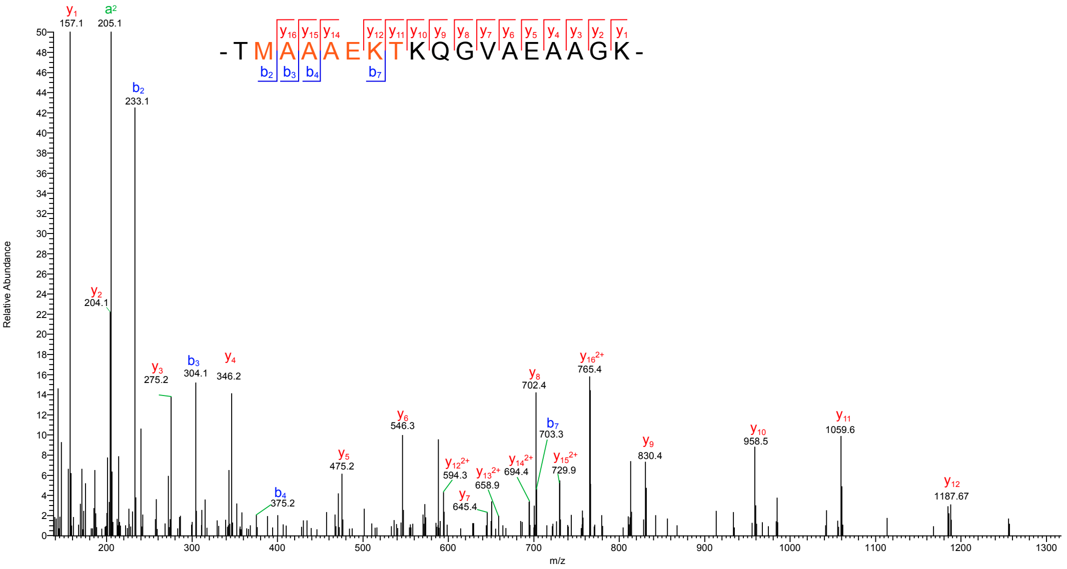

b

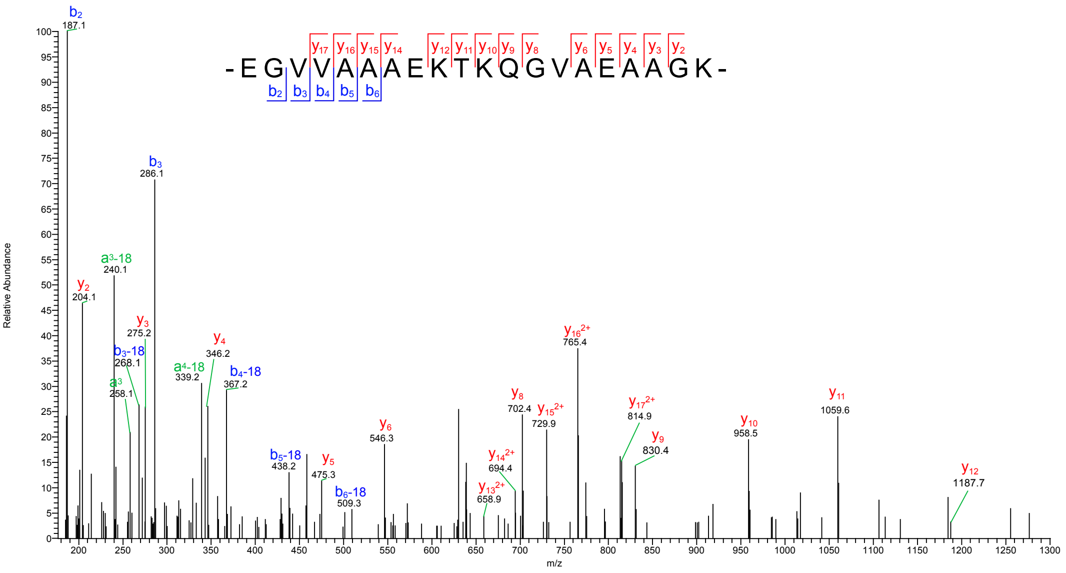

Figure S1 Identification of wild-type and mutant α-synucleins from JOS by mass spectrometry.

Peptide sequences are shown at the top of each panel, together with collision-induced fragmentation patterns; b ions (N-terminal) and y ions (C-terminal) are generated during peptide fragmentation. Doubly charged ions are labelled “2+”. The ions that have a neutral loss are shown in green. Pyroglutamate-modified ions are labelled “-18”.

a, MS/MS spectrum of mutant α-synuclein sequence TMAAAEKTKQGVAEAAGK. The insertion sequence is shown in orange.

b, MS/MS spectrum of wild-type α-synuclein sequence EGVVAAAEKTKQGVAEAAGK.

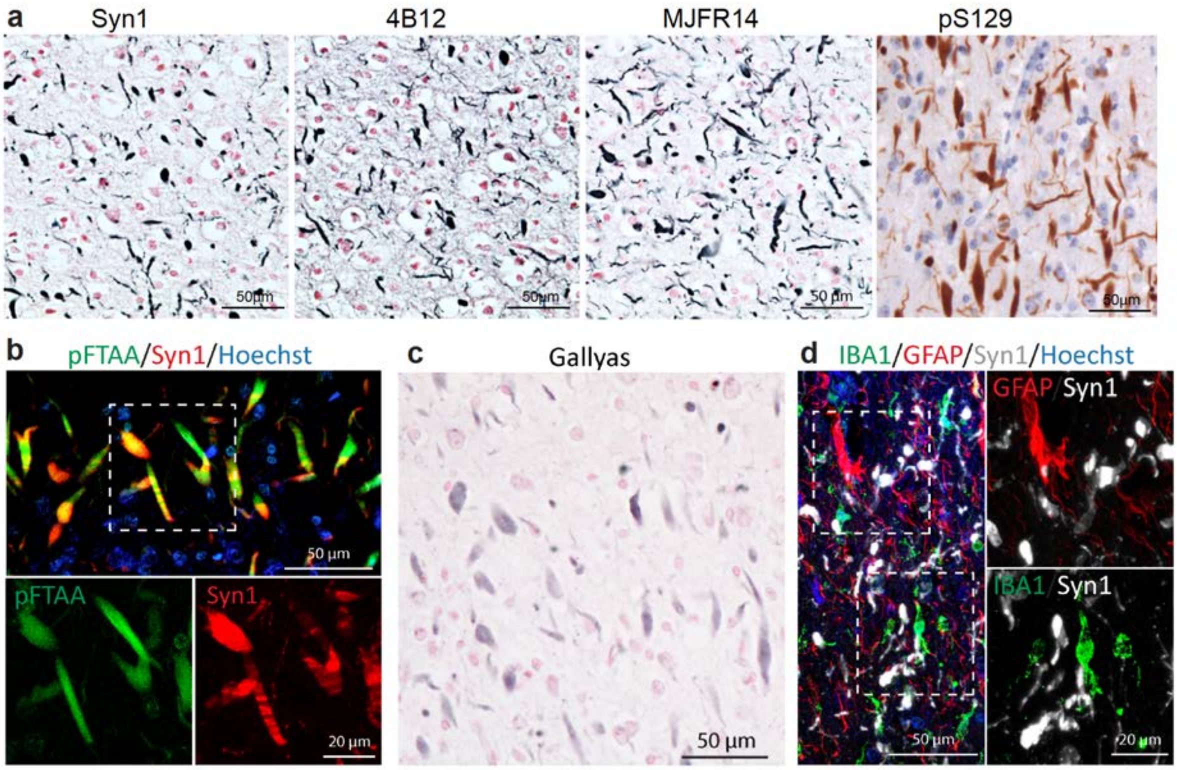

Figure S2 Staining of α-synuclein inclusions from frontal cortex of JOS.

a, Immunostaining for α-synuclein using antibodies Syn1, 4B12 and MJFR14, showing numerous stained inclusions (blue). Nuclei are stained red. Using antibody pS129, numerous inclusions (brown) are seen in nerve cell processes. Nuclei are stained blue. Diaminobenzidine was used as the chromogen for antibody pS129.

b, Double-labelling immunofluorescence using anti-α-synuclein antibody Syn1 (red) and luminescent conjugated oligothiophene pFTAA (green). Double labelling (yellow). Nuclei are stained blue (Hoechst dye).

c, Gallyas-Braak silver staining (black). Nuclei are stained red.

d, Triple-labelling immunofluorescence using anti-α-synuclein antibody Syn1 (white), anti-microglial marker Iba1 (green) and anti-astrocyte marker GFAP (red). Nuclei are stained blue (Hoechst dye).

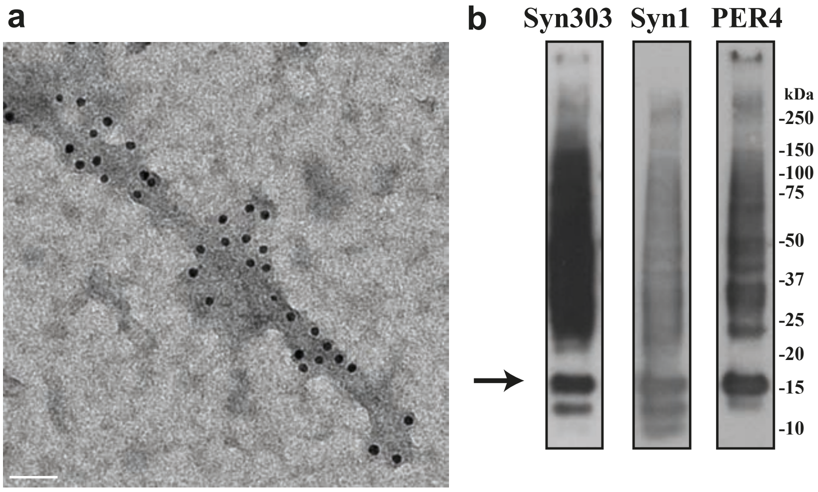

Figure S3

Negative-stain immunoelectron microscopy and immunoblotting of α-synuclein filaments from frontal cortex of JOS

a, Negative-stain immunoelectron microscopy of JOS filaments, with polyclonal antibody PER4.

b, Immunoblotting of JOS filaments. Sarkosyl-insoluble material was blotted with monoclonal antibodies Syn303 and Syn1, and polyclonal antibody PER4. The arrow points to monomeric α-synuclein.

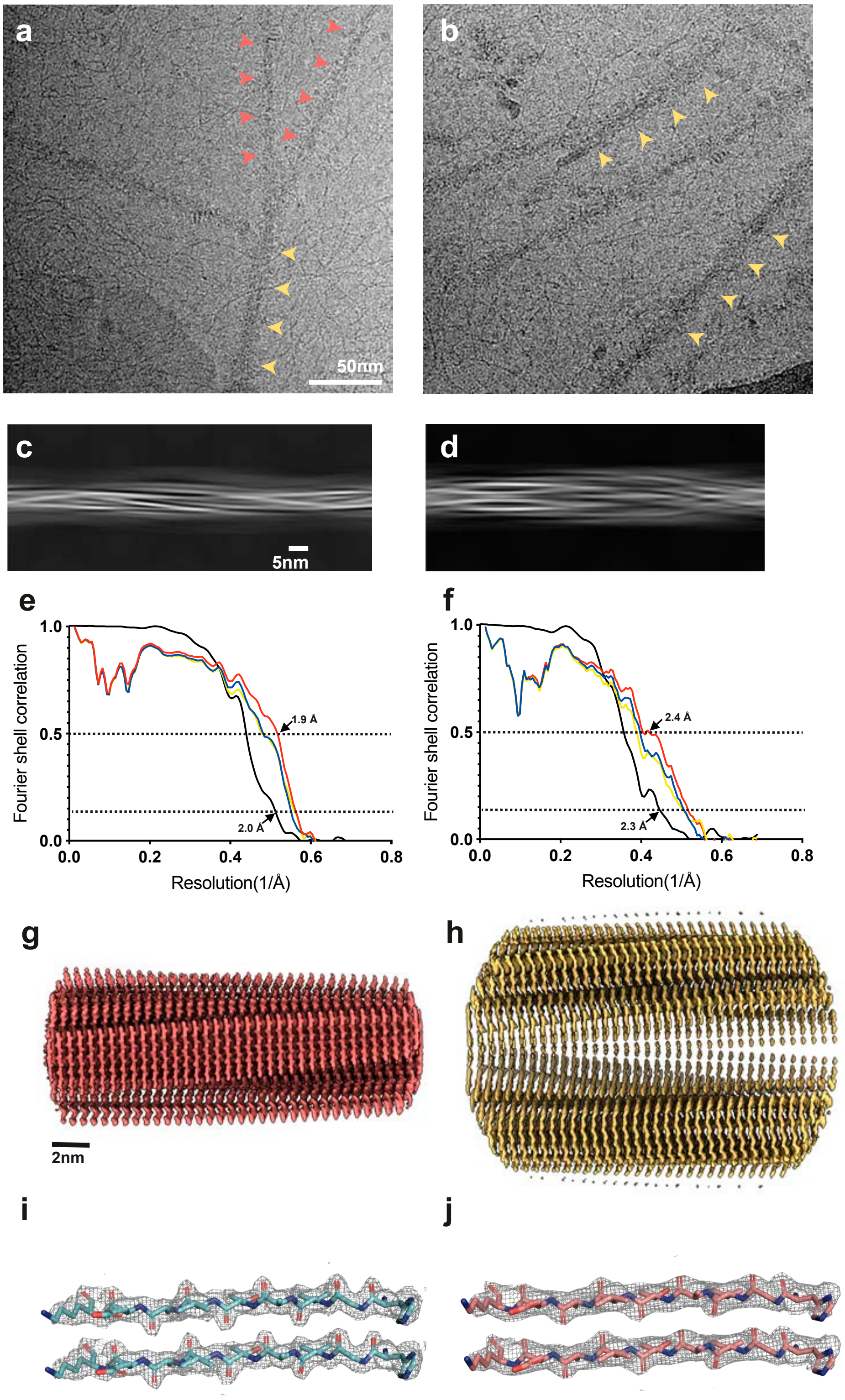

Figure S4 Cryo-EM images and resolution estimates of α-synuclein filaments from the frontal cortex of JOS.

a,b, Orange arrowheads (a) point to singlet, yellow arrowheads (a and b) to doublet JOS filaments. Scale bar, 50 nm.

c,d, Projection features of singlet (c) and doublet (d) JOS filaments. Scale bar, 5 nm.

e,f, Fourier shell correlation (FSC) curves for cryo-EM maps and structures of α-synuclein filaments in JOS singlet (e) and doublet (f) filaments. FSC curves for two independently refined cryo-EM half maps are shown in black; for the final refined atomic model against the final cryo-EM map in red; for the atomic model refined in the first half map against that half map in blue; for the refined atomic model in the first half map against the other half map in yellow.

g,h, Side views of JOS singlet (g) and doublet (h) filaments.

i,j, Zoomed-in views of the main chains of singlet (i) and doublet (j) filaments, showing the densities of main-chain oxygen atoms.

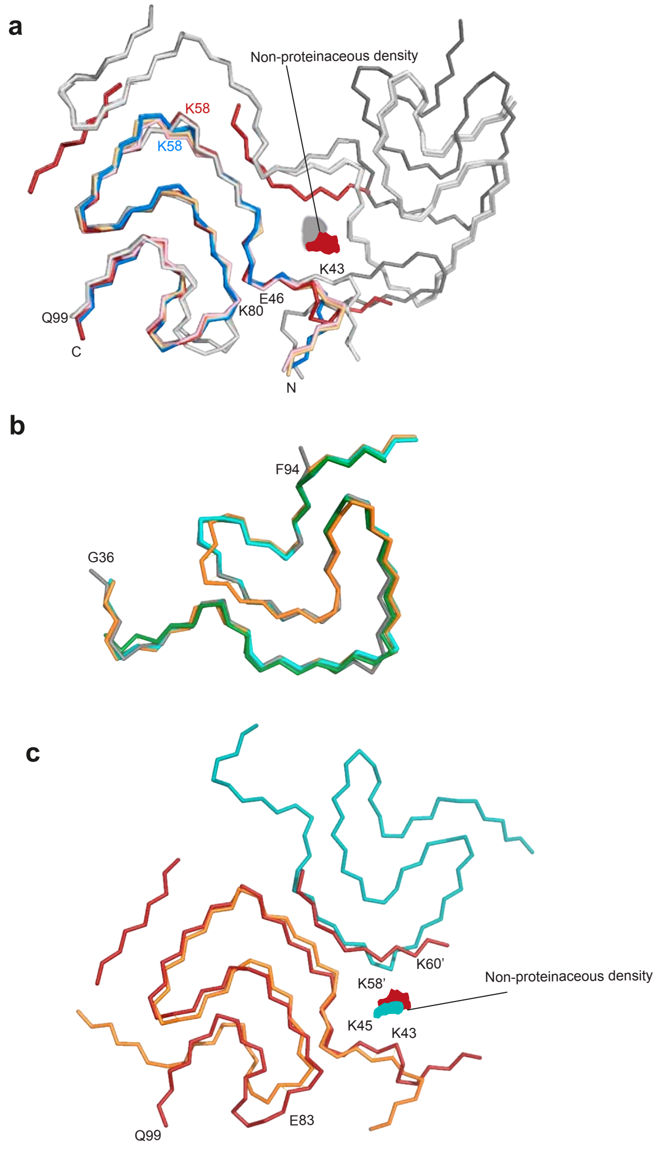

Figure S5 Comparison of α-synuclein folds.

a, Superposition of the JOS fold (red) onto the MSA folds (PDB:6XYO; PDB:6XYP; PDB:6XYQ)(grey); onto the fold from seeded assembly using MSA brain seeds (PDB:7NCK) (pink); onto assembled recombinant H50Q α-synuclein (PDB:6PEO) (yellow) and onto assembled recombinant A53T α-synuclein (PDB:6LRO) (blue). Putative cofactor densities are shown in red for JOS and in grey for MSA.

b, Superposition of the protofilament folds from the mixture [protofilament A of Type 1 (cyan), protofilament B of Type 1 (orange) and protofilament of Type 2 (green)] onto the core of MSA protofilament IA, residues G36-F94 (PDB:6XYO) (grey).

c, Superposition of the JOS fold on the structure of dimeric filaments of Type 1A, based on residues K43-E83 of JOS and protofilament B. Type 1A in cyan and Type 1B in orange. Putative cofactor densities are shown in red for JOS and in cyan for the Type 1 mixture.

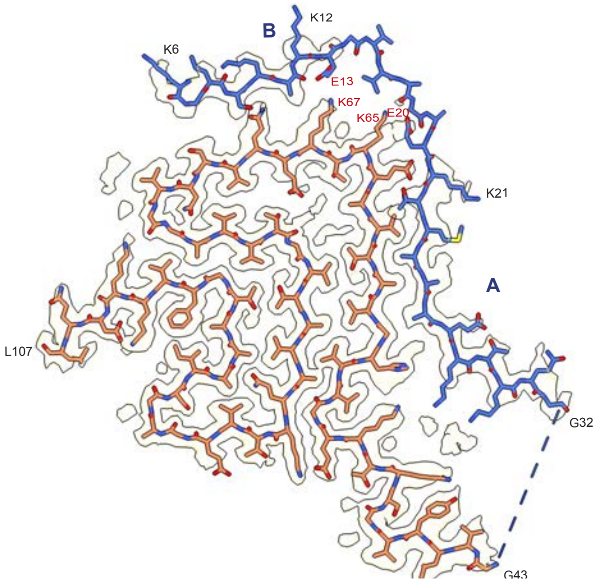

Figure S6 Model of the JOS fold with mutant α-synuclein.

Island B is assigned to the sequence $_{6}$KGLSKAK$_{12}$ and connected to island A ($_{21}$KTMAAAEKTKQG$_{32}$), both in blue. Core regions are in orange and map densities in transparent grey. Residues forming internal salt bridges are labelled red.

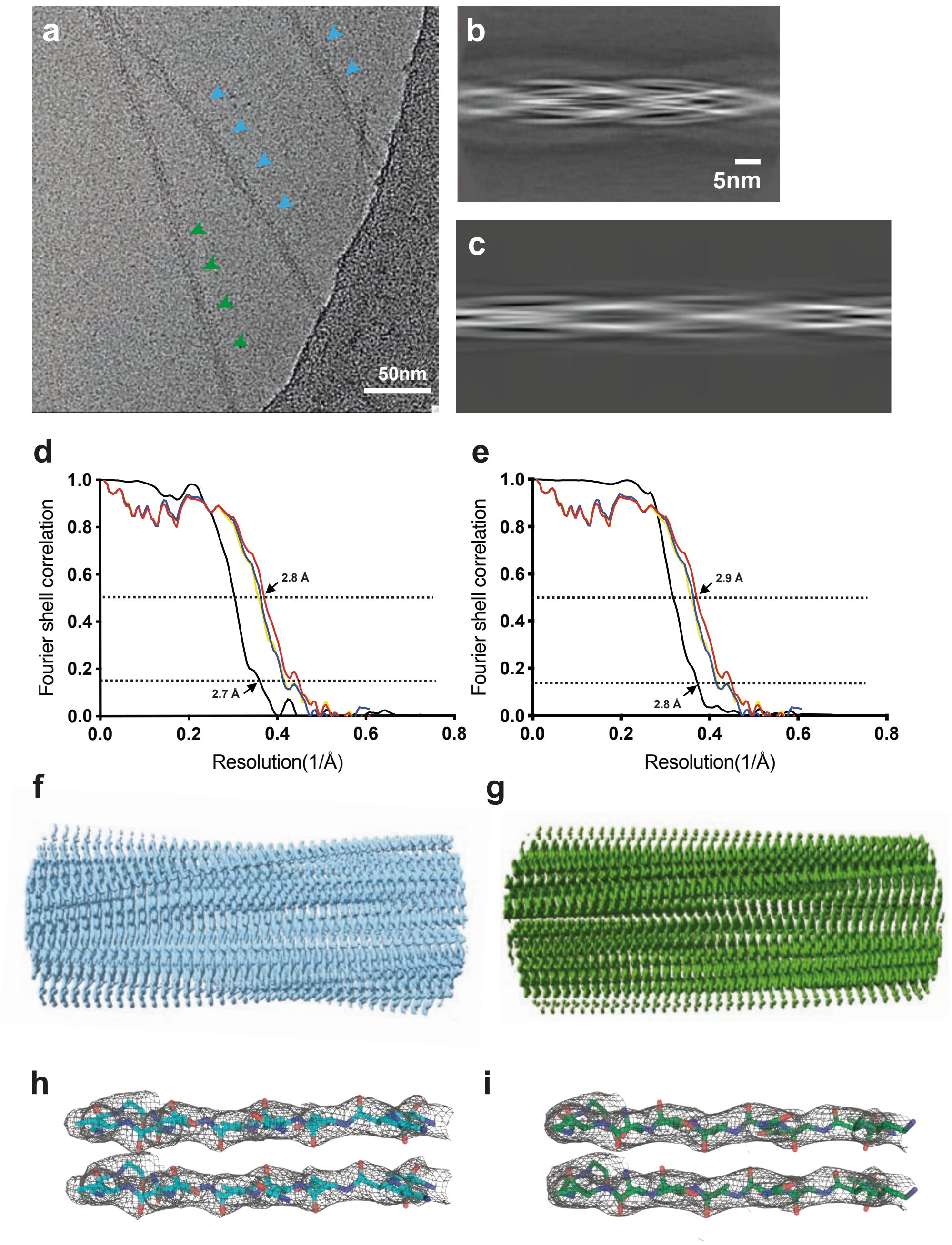

Figure S7 Cryo-EM images and resolution estimates of α-synuclein filaments assembled from recombinant proteins.

a, Wild-type and mutant □-synuclein filaments. Cyan arrowheads point to Type 1 and green arrowheads to Type 2 filaments. Scale bar, 50 nm.

b,c, Projection features of Type 1 (b) and Type 2 (c) α-synuclein filaments. Scale bar, 5 nm.

d,e, Fourier shell correlation (FSC) curves for cryo-EM maps and structures of Type 1 (d) and Type 2 (e) filaments. FSC curves for two independently refined cryo-EM half maps are shown in black; for the final

refined atomic model against the final cryo-EM map in red; for the atomic model refined in the first half map against that half map in blue; for the refined atomic model in the first half map against the other half map in yellow.

f,g, Side views of Type 1 (f) and Type 2 (g) α-synuclein filaments.

h,i, Zoomed-in views of the main chains of Type 1 (h) and Type 2 (i) filaments, showing the densities of main-chain oxygen atoms.
